# Inflammation and limited adaptive immunity predict worse outcomes on immunotherapy in head and neck cancer

**DOI:** 10.1038/s41698-025-01020-6

**Published:** 2025-08-05

**Authors:** Lisa Paschold, Christoph Schultheiss, Paul Schmidt-Barbo, Konrad Klinghammer, Dennis Hahn, Mareike Tometten, Philippe Schafhausen, Markus Blaurock, Anna Brandt, Ingunn Westgaard, Simone Kowoll, Alexander Stein, Axel Hinke, Mascha Binder

**Affiliations:** 1 Department of Internal Medicine IV, Oncology/Hematology, Martin-Luther-University Halle-Wittenberg, Halle (Saale), Germany; 2 Division of Medical Oncology, University Hospital Basel, Basel, Switzerland; 3 Laboratory of Translational Immuno-Oncology, Department of Biomedicine, University and University Hospital Basel, Basel, Switzerland; 4 Collaborative Research Institute Intelligent Oncology (CRIION), Freiburg, Germany; 5 Department of Hematology and Oncology, Campus Benjamin Franklin, Charité—Universitätsmedizin Berlin, Corporate Member of Freie Universität Berlin, Humboldt-Universität zu Berlin, and Berlin Institute of Health, Berlin, Germany; 6 Department of Hematology, Oncology, Stem-Cell Transplantation and Palliative Care, Klinikum Stuttgart, Stuttgart, Germany; 7 Department of Hematology, Oncology, Hemostaseology and Stem Cell Transplantation, Medical Faculty, RWTH Aachen University, Aachen, Germany; 8 Department of Oncology, Hematology and Bone Marrow Transplantation with Section of Pneumology, University Medical Center Hamburg-Eppendorf, Hamburg, Germany; 9 Department of Otorhinolaryngology, Head and Neck Surgery, University Medicine Greifswald, Greifswald, Germany; 10 Ultimovacs, Oslo, Norway; 11 Koordinierungszentrum für Klinische Studien Halle (KKS), Medical Faculty, Martin-Luther-University Halle-Wittenberg, Halle (Saale), Germany; 12 Hematology-Oncology Practice Eppendorf (HOPE), Hamburg, Germany; 13 Clinical Cancer Research Consulting (CCRC), Düsseldorf, Germany

**Keywords:** Cytokines, Head and neck cancer, Adaptive immunity, Lymphocytes, Tumour immunology, Cancer, Immunology, Biomarkers, Medical research, Oncology

## Abstract

Most patients with relapsed or metastatic head and neck squamous cell carcinoma (rmHNSCC) do not experience durable responses to PD-1 immune checkpoint inhibitors. PD-L1 tissue expression is the most commonly assessed response marker, but an insufficient predictor of treatment outcome. To identify suitable response biomarkers, we profiled the FOCUS trial (Registered at ClinicalTrials.gov: NCT05075122) cohort for several blood- and tissue-based markers. PD-L1 levels in the tumor or tumor microenvironment were not associated with treatment benefit. In contrast, inflammation-related markers such as IL-6, sCD25, and sTIM-3, as well as high peripheral neutrophils, cell-free DNA levels, and T cell receptor repertoire clonality, were associated with poor clinical outcomes. Patients lacking these high-risk markers performed remarkably well on inhibition of immune checkpoints with pembrolizumab. Biomarker-guided patient selection for pembrolizumab monotherapy or novel combinatorial approaches—potentially including anti-inflammatory agents—for patients with immune-impaired, inflammatory profiles may be the next step in personalizing immunotherapy for these hard-to-treat patients.

## Introduction

Head and neck squamous cell carcinomas (HNSCC), primarily originating from the squamous epithelium of the oral cavity, pharynx, and larynx, rank as the sixth most prevalent cancer globally, with nearly 900,000 new cases and ~45,000 deaths per year^1–4^. HNSCC predominantly affects males and is causally linked to tobacco and alcohol consumption as well as infection with human papillomavirus (HPV) strains 16 and 18^1,2,5–7^. Early-stage locoregional disease can often be cured, but more than half of these cases relapse, and around 15-30% develop metastatic disease (rmHNSCC)^1,8,9^. Survival time for patients with rmHNSCC has doubled over the past decade, primarily due to advances in systemic treatment^1,4,10–12^. Despite this progress, the median overall survival (OS) for these patients remains limited to 12–14 months^12,13^.

Preclinical data indicate that HNSCC is highly immunosuppressive, marked by abnormal proinflammatory cytokine secretion and impaired immune effector cell function^14,15^. Therefore, one of the major advances in systemic treatment of rmHNSCC was the introduction of immunotherapies. Landmark phase III trials have led to the approval of two anti-programmed cell death-1 (PD-1) antibodies—pembrolizumab and nivolumab—for use in this indication^16,17^. More recently, pembrolizumab also received approval as a first-line treatment with or without concomitant chemotherapy^18^. Despite these advancements, only a subset of patients with rmHNSCC benefit from immunotherapy, underscoring the urgent need to identify novel biomarkers to optimize treatment strategies. PD-L1 positivity measured as combined positive score (CPS; The number of PD-L1 positive cells relative to all cells in a given section) is currently the only predictive biomarker for response to immune checkpoint inhibitors in rmHNSCC in routine clinical practice, but there is still an important subset of patients with PD-L1 positive disease that does not derive benefit from these drugs^19,20^.

In this study, we conducted comprehensive biomarker analyses using biomaterial from patients enrolled in the FOCUS trial, all of whom received pembrolizumab^21^. Our objective was to identify simple, clinically applicable markers to predict the outcome of checkpoint inhibitor treatment. Our findings demonstrate that patients with a high-inflammation and impaired T cell profile experience suboptimal outcomes with immune checkpoint inhibition alone, indicating the need for additional or alternative therapeutic approaches.

## Results

### Patient characteristics, study treatment, and survival outcomes in the cohort

The FOCUS trial enrolled 75 evaluable patients from August 2021 to July 2023. 25 were enrolled into the calibration arm receiving pembrolizumab monotherapy and 50 into an experimental arm receiving pembrolizumab in combination with the hTERT vaccine UV1. Detailed patient characteristics are described in ref. ^21^. The study population was representative for patients with rmHNSCC in that it also included 18% of patients with an ECOG performance score of 2.

**Fig. 1:**
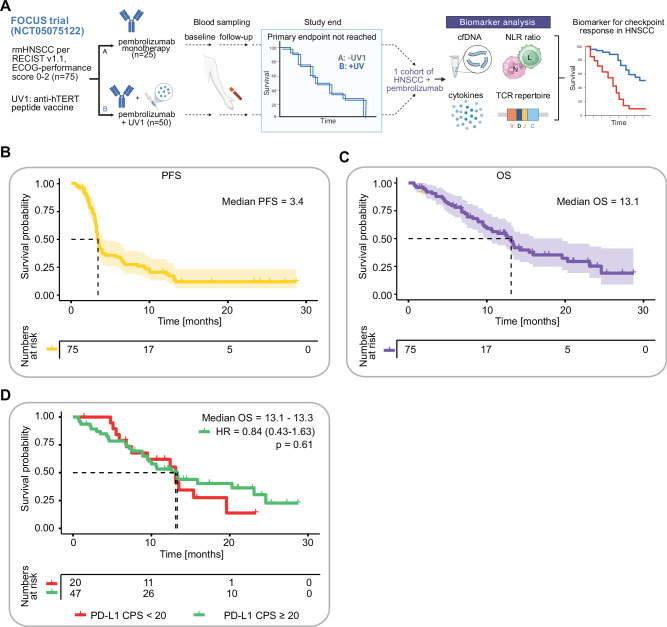
Efficacy of pembrolizumab (±UV1) treatment in patients with rmHNSCC and as a function of PD-L1 CPS in the FOCUS trial. **A** Schematic workflow of the FOCUS study and the performed biomarker analysis. Kaplan–Meier estimates of progression-free (**B**) and overall survival (**C**), and according to PD-L1 CPS subgroup (cut-off 20) in patients with completed PD-L1 screening (**D**). HR hazard ratio. Statistic: log-rank test.

The trial did not meet its primary endpoint^21^. UV1 treatment did not result in higher-than-expected rate of progression-free survival (PFS) at 6 months. Therefore, we combined both pembrolizumab-containing study arms for the biomarker analysis reported here (Fig. 1A). To avoid any potential biases from the experimental UV1 vaccination, all analyses were additionally plotted by treatment arm as shown in the supplementary data section. PFS and overall survival (OS) are shown in Fig. 1B, C. With a median follow-up of 11.3 months, the median PFS and OS were very comparable to the pembrolizumab arm of the KEYNOTE-048 trial that had enrolled only patients up to an ECOG performance score of 1^18^. The median PFS of 3.4 months compared to 2.3 months for patients with PD-L1 CPS 1 or more and 3.4 months for patients with PD-L1 CPS 20 or more in the KEYNOTE-048 trial^18^. The median OS of 13.1 months compared to 13.0 months in the KEYNOTE-048 trial^18^.

### Efficacy as a function of PD-L1 CPS

PD-L1 expression is an established prognostic biomarker in HNSCC^22,23^. Interestingly, in the PD-L1 positive population studied in the FOCUS trial, high or low PD-L1 combined positivity scores (PD-L1 CPS below or above 20) did not appear to segregate patient subsets with higher or lower clinical benefit (Fig. 1D and Supplementary Fig. 1A, B).

### T cell receptor repertoire profiling

**Fig. 2:**
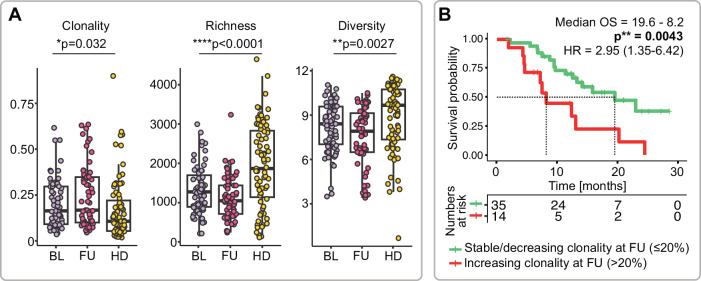
T cell receptor repertoire metrics in HNSCC patients treated with pembrolizumab on the FOCUS trial. **A** Blood T cell repertoire clonality, richness, and diversity in healthy individuals (HD; n = 78) and the HNSCC patient population with available TCR data at baseline assessment (BL, n = 64) and before the second treatment cycle (FU; n = 57) of the FOCUS trial. Statistics: ordinary one-way ANOVA. **B** Overall survival (OS) outcomes of patients with increasing T cell restriction (>20% increase in T cell receptor clonality over the first pembrolizumab treatment cycle). HR hazard ratio. Statistics: log-rank test. *p ≤ 0.05, **p ≤ 0.01,***p ≤ 0.001, ****p ≤ 0.0001.

Previous studies from our group had shown that T cell repertoire metrics, as indicators of an individual’s immune system composition and capacity to mount a tumor-specific response, may be a strong predictive biomarker for immune checkpoint blockade in distinct disease settings^24–28^. We assessed T cell immune repertoires by next-generation sequencing of the T cell receptor beta (TRB) locus from patient blood at baseline (BL) and prior to the second pembrolizumab dose in both arms (follow-up; FU). As controls, we used a selection of age-matched healthy controls from previous studies (n = 78; 54 male, 24 female; median age: 59, range 44–87)^29–31^. Patient immune repertoires showed differences in global T cell immune metrics to healthy age-matched control individuals (Fig. 2A). While BL T cell metrics did not appear to determine outcomes on pembrolizumab (data not shown), the dynamics of T cell repertoire clonality correlated with overall survival. Patients with increasing T cell repertoire restriction (increase of T cell repertoire clonality >20% above BL) showed unfavorable outcomes on pembrolizumab compared to patients with stable repertoires (Fig. 2B and Supplementary Fig. 2A, B).

### Blood soluble factor analysis

**Fig. 3:**
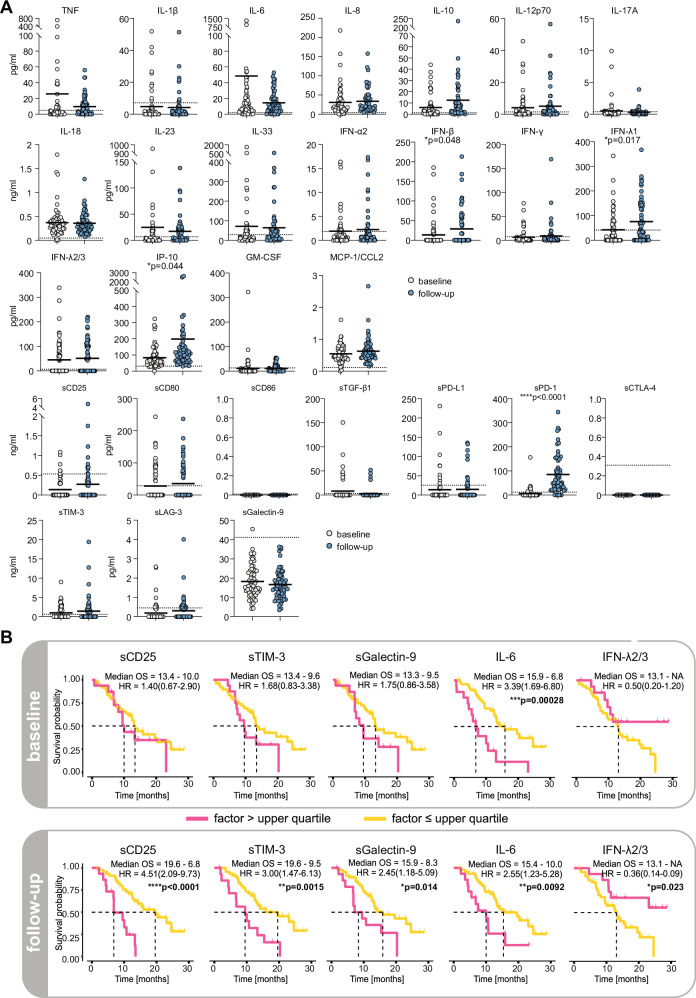
Soluble factor and chemokine profiling baseline and upon pembrolizumab treatment. **A** Circulating immune checkpoint molecules and cytokines in patients from the FOCUS trial at baseline assessment (BL; n = 65) and before the second cycle of pembrolizumab (FU; n = 61). Dashed lines indicate means from 20 randomly chosen healthy donors. Statistics: unpaired two-tailed t-test. **B** Overall survival (OS) outcomes on pembrolizumab in patients with elevated levels of circulating immune checkpoint molecules and cytokines (upper quartile) as compared to the rest of the cohort at BL and on treatment. HR hazard ratio. Statistics: log-rank test. *p ≤ 0.05, **p ≤ 0.01,***p ≤ 0.001, ****p ≤ 0.0001.

HNSCC is marked by abnormal proinflammatory cytokine secretion^32^. As a proxy of the degree of inflammation, we assessed cytokine patterns in the blood of our patients. Moreover, we were interested in the levels of circulating checkpoint molecules, some of which had been found to counteract immune checkpoint inhibition in other disease settings^33,34^. Blood testing was performed at BL and before the second treatment cycle (follow-up, FU). Twenty randomly selected healthy individuals were used as controls^35^. As shown in Fig. 3A, B, most of the analyzed inflammatory factors displayed high plasma levels at both sampling time points as compared to healthy individuals. Notable exceptions were IL-1β, IL-17A, IFN-γ, and GM-CSF (Fig. 3A). While mean levels of TNF, IL-6, and IL-23 trended towards lower levels at FU, IL-10, IFN-β, and IP-10 showed the opposite pattern (Fig. 3A). IFN-λ1 and IFN-α2 were only elevated at FU but not at BL time points (Fig. 3A).

In contrast, plasma levels of most soluble immune checkpoints were actually diminished compared to the levels measured in the 20 healthy blood donors (sCD25, sPD-L1, sCTLA-4, sLAG-3 and sGalectin-9; Fig. 3A). Exceptions were sTIM-3 which was slightly elevated at both time points and sPD-1 which was highly elevated after only one dose of the PD-1-directed checkpoint inhibitor at FU (Fig. 3A). Next, we assessed the clinical courses in patients with high levels of selected soluble factors. Individuals with levels in the upper quartile were considered high level. When testing these patients against the rest, we observed that patients with high cytokine plasma levels had a lower survival probability on pembrolizumab as compared to patients with lower levels (Fig. 3B). This was especially true for patients with elevated levels of sCD25, sTIM-3, IL-6 and sGalectin-9 and more pronounced after the first dose of the immune checkpoint inhibitor (FU) than at BL assessment (Fig. 3B; Supplementary Fig. 3A, B). Interestingly, this was reversed in patients with high IFN-λ2/3 levels (Fig. 3B). Notably, the beneficial effect of high IFN-λ2/3 levels was mainly driven by samples from the initial study arm A (pembrolizumab only), which implies a potential effect of the vaccination approach on IFN-λ2/3 plasma levels (Supplementary Fig. 3).

### Cell-free DNA levels and neutrophil-to-lymphocyte ratios (NLR)

**Fig. 4:**
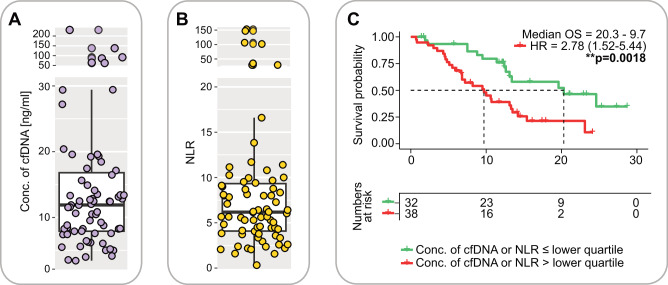
Median cell-free DNA (cfDNA) and neutrophil-to-lymphocyte ratios (NLR) in patients with HNSCC treated with pembrolizumab on the FOCUS trial. **A** cfDNA levels and **B** NLR in HNSCC patients (n = 70) from the FOCUS trial at baseline assessment. **C** Overall survival (OS) in patients with low cfDNA and/or NLR (lower quartile). HR hazard ratio. Statistics: log-rank test. *p ≤ 0.05, **p ≤ 0.01,***p ≤ 0.001, ****p ≤ 0.0001.

In the inflammatory context of cancer, high levels of DNA seem to be released into the blood, resulting in elevated cell-free DNA (cfDNA) levels in cancer patients as compared to healthy individuals. The majority (roughly 75%) of this DNA has been found to derive from neutrophils rather than from the tumor itself or its immediate environment^36^. Given the inflammatory signature of HNSCC in general and the unfavorable outcomes on immune checkpoint inhibition observed with this signature in our FOCUS trial, we wished to analyze cfDNA levels along with the NLR as potentially predictive biomarkers. We reasoned that lower cfDNA levels and lower NLR may define a patient subpopulation that might benefit from pembrolizumab monotherapy without the need for additional, e.g., chemotherapy. cfDNA levels of normal individuals have been shown to have cfDNA levels around 4.3 ng/ml, while patients with stage I-III cancer have levels around 12.6 ng/ml^36^. In line with this, our patients had elevated cfDNA levels with a median of 11.9 ng/ml (Fig. 4A).

Also, NLR were elevated in our cohort (median of 6.18) as compared to healthy individuals that typically have a ratio of 1–3 (Fig. 4B). Patients with cfDNA in the lower quartile of our cohort (below 7.9 ng/ml) and/or NLR in the lower quartile (below 4.1) had favorable clinical courses on pembrolizumab (Fig. 4C and Supplementary Fig. 4A, B). Yet, these two features—cfDNA levels and NLR—did not show any obvious correlation (Table 1).

**Table 1:** Patient subsets from the FOCUS trial displaying high or low cfDNA and/or NLR at baseline assessment

	cfDNA high	cfDNA low	
NLR high	38	15	53
NLR low	15	2	17
	53	17	70

### Immunotypes and biomarker importance tested in the FOCUS trial

**Fig. 5:**
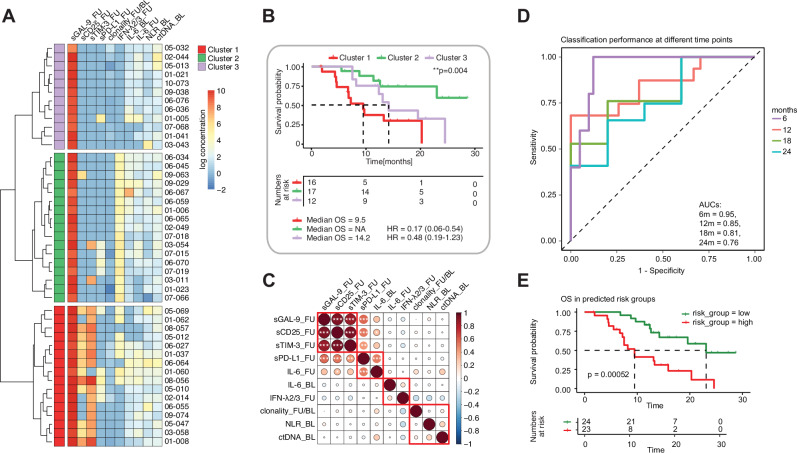
Unsupervised cluster analysis to determine immunotypes of patients with HNSCC on pembrolizumab treatment. **A** Unsupervised hierarchical clustering (Ward D2 method, Canberra distance) of log10 transformed cytokine concentrations in plasma samples of indicated patients from the FOCUS trial (n = 45). **B** Survival analysis for the three patient clusters as established in (A). OS overall survival, HR hazard ratio. Statistics: log-rank test. **C** Correlation of parameters studied in (**A**) in a correlation matrix. Asterisks indicate significant correlations. **D** Receiver operating characteristic (ROC) curve of prognostic performance of the Cox model built on biomarker data. AUC area under the curve, m months. **E** Overall survival (OS) in patients predicted as high risk or low risk according to the Cox model. Statistics: log-rank test. *p ≤ 0.05, **p ≤ 0.01,***p ≤ 0.001, ****p ≤ 0.0001.

We performed a broader unsupervised cluster analysis in all patients who had matched and complete BL and FU data for all analyzed factors (TCR, NLR, cfDNA, cytokines/soluble factors) to understand potential immunotypes beyond individual markers. All biomarkers that had shown correlation with OS in the individual analyses were included. We identified essentially three clusters (Fig. 5A): One group of patients was mainly characterized by secretion of the soluble immune molecules sTIM-3 and sCD25 at FU (red), another group showed high levels of IFN-λ2/3 at FU (green), and a third group showed neither of these features (violet). Overall survival was short in the sTIM-3/sCD25 group, long in the IFN-λ2/3 group and intermediate in the group without these features (Fig. 5B). A comprehensive analysis including all of these biomarkers suggested a positive correlation of the sTIM-3/sCD25 cluster with high sGalectin-9 and—to a lesser extent—with sPD-L1 levels (Fig. 5C). There was some correlation of sPD-L1 levels with IL-6 levels measured at FU. All other biomarkers tested did not show significant correlation with this or other clusters.

To individually assess the combined predictive power of biomarkers associated with OS, we next performed a multivariate Cox regression. To address feature correlation and reduce overfitting, we applied Lasso regularization. Using a 3-fold cross-validation approach, the model selected four biomarkers with non-zero coefficients from the initial set of 10 (Fig. 5A, C): sCD25_FU, sTIM-3_FU, IL-6_FU, and clonality_FU/BL, highlighting their collective association with OS. These features were then selected to refit an unpenalized model for interpretability. In this model, clonality_FU/BL and IL-6_FU were significantly associated with reduced survival, whereas sCD25_FU, sTIM-3_FU were not. Given the observed correlation between sCD25_FU, sTIM-3_FU (Fig. 5C), we refined the model by removing sTIM-3_FU, resulting in a more stable and interpretable final model. This final model achieved a concordance index (C-index) of 0.774, indicating good discriminatory ability. All three biomarkers were significantly associated with reduced OS: sCD25_FU (HR = 1.001, 95% CI: 1.000–1.002, p = 0.007), IL-6_FU (HR = 1.040, 95% CI: 1.002–1.080, p = 0.042), and clonality_FU/BL (HR = 2.056, 95% CI: 1.415–2.988, p < 0.001). These findings are consistent with trends observed in earlier univariate analysis.

Finally, we computed time-dependent ROC curves based on predicted risk scores at 6, 12, 18, and 24 months to evaluate the prognostic performance of our final Cox model. Notably, this analysis was restricted to 47 patients with complete data availability. Nevertheless, the model achieved AUCs of 0.95, 0.85, 0.81 and 0.76, respectively, indicating a good predictive and relatively stable discriminatory performance over time (Fig. 5D). Furthermore, Kaplan–Meier analysis of patients stratified into predicted high and low risk revealed a significant difference in OS between groups (p < 0.001), further supporting the prognostic utility of the findings (Fig. 5E).

## Discussion

Immune checkpoint inhibitors have significantly expanded the treatment options for rmHNSCC. However, the ideal treatment schedules and combination partners are still uncertain. In fit patients with relapsed or refractory PD-L1-positive HNSCC, pembrolizumab as a monotherapy or in combination with chemotherapy has demonstrated comparable effectiveness in broad, unselected cohorts^13,17^. It is likely that specific patient subgroups benefit more from the addition of chemotherapy, while others may achieve sufficient responses with pembrolizumab alone. Furthermore, certain patients may require completely different therapeutic approaches altogether. This question is critical, as head and neck cancer patients often present with comorbidities, and the addition of chemotherapy can significantly increase treatment-related toxicities.

In our study, we treated a cohort of patients with up to and including ECOG 2 performance status using pembrolizumab. In the study arm B, patients received an hTERT vaccine in addition to the pembrolizumab backbone, but this combination failed to meet the primary endpoint of PFS and was therefore deemed ineffective^21^. Nonetheless, we identified several intriguing biomarkers in this cohort that correlate with clinical outcomes. These markers were associated with inflammatory processes (e.g., elevated cytokine levels, high cfDNA concentrations, increased neutrophil-to-lymphocyte ratios) or impaired T cell immunity (e.g., high T cell repertoire clonality, which likely indicates a lack of sufficient repertoire breadth to mount a strong anti-tumor response, secretion of immune checkpoint molecules). Multivariate modeling identified sCD25, IL-6, and TCR clonality as the most robust correlates of OS. Patients exhibiting a pronounced inflammatory profile or restricted T cell repertoire with high immune checkpoint molecule secretion experienced poor outcomes on pembrolizumab, similar to observations in colorectal end esophagogastric cancer patients under checkpoint blockade^24,27,28,37^. However, due to the study design, it remains unclear whether these factors are predictive or merely prognostic in HNSCC.

Nevertheless, their detected activity provides some mechanistic insights underlying the observed pembrolizumab efficacy. Elevated IL-6, commonly found in HNSCC, is known to promote JAK/STAT-dependent tumor cell proliferation^38^. Chronic IL-6 exposure has also been linked to T cell exhaustion and resistance to checkpoint blockade^39–41^. Interestingly, our data indicate that increased levels of IFN-λ2/3—a type III interferon which also signals via the JAK/STAT pathway—are associated with improved pembrolizumab efficacy, even in the presence of elevated IL-6. This may be explained by IFN-λ-mediated upregulation of MHC-I^42^ and PD-L1/PD-L2^43^, enhancing tumor visibility and responsiveness to PD-1 blockade. The fact that expression of the IFN-λ2/3 receptor (IFNLR1) is mostly restricted to epithelial cells, mucosal surfaces, and dendritic cell subsets also highlights the potential relevance of this signaling axis in HNSCC^44^. Importantly, the beneficial effects of IFN-λ2/3 were primarily observed in patients from study arm A (pembrolizumab only), suggesting that UV1 vaccination may influence IFN-λ2/3 plasma levels. This hypothesis is particularly compelling given that UV1 is designed for uptake by dendritic cells^21^, key responders to type III interferons at barrier surfaces. In addition, we found that patients with high levels of sCD25 and sTIM-3 exhibited poor survival on PD-1 blockade. Although the precise biology of these two soluble markers remains incompletely understood, both have been associated with impaired T cell effector function and resistance to immune checkpoint blockade^45,46^, highlighting the prominent multifactorial role of T cell immune regulation for checkpoint efficacy.

The role of inflammation in HNSCC as a resistance mechanism to immunotherapy, or as a direct driver of tumor progression, remains unresolved^14,15^. This observation raises the important question of whether combining immunotherapy with chemotherapy or other agents, such as anti-inflammatory drugs, could enhance treatment efficacy and improve patient outcomes. One potential approach could involve the combination of immune checkpoint inhibitors with JAK inhibitors, which target signaling pathways downstream of interferon and type 1 cytokine receptors on immune cells. Recent studies have shown success with this concept in non-small cell lung cancer and Hodgkin’s lymphoma^47,48^. In these models, JAK inhibition with ruxolitinib or itacitinib reversed CD8^+^ T cell exhaustion driven by myeloid-derived suppressor cell (MDSC)-mediated IFN-JAK/STAT signaling. While MDSCs are also present in HNSCC tumors, their infiltration is not yet linked to disease etiology, though infiltration rates do correlate with clinical stage^49^. Notably, elevated NLRs have been associated with higher MDSC frequencies and worse prognosis across several cancer types^50–53^.

Furthermore, JAK inhibition could offer the added benefit of direct anti-tumor activity, given the reliance of many HNSCC tumors on JAK-STAT signaling pathways^54^. This dual-action approach—targeting both immune modulation and tumor signaling—holds promise for improving outcomes in this challenging patient population. However, it is important to recognize that disruptions in JAK-STAT signaling, or the acquisition of loss-of-function mutations in this pathway, are well-established mechanisms of resistance to immune checkpoint blockade^55^. In addition, JAK-STAT signaling plays a crucial role in maintaining T cell survival and function^56^. Thus, any potential therapeutic benefit from JAK inhibition must be carefully weighed against the risk of impairing T cell-mediated immunity, and further validation is needed to support its use alongside PD-1 inhibitors in a given setting.

In the context of this complex immune environment, our findings also emphasize the limitations of relying on single biomarkers like CPS to predict response to immune checkpoint blockade. Although all patients in the FOCUS study had CPS ≥ 1, the quantitative CPS value showed no association with outcome. This may reflect confounding by intratumoral heterogeneity, dynamic regulation of PD-L1 expression, or insufficient T cell infiltration. Similarly, cfDNA is not a categorical marker but may reflect a mix of tumor burden, systemic inflammation, and treatment response. The weak correlation between cfDNA and NLR in our cohort underscores this complexity.

Given that neutrophils are a dominant source of cfDNA^36^, the data may suggest a greater relative contribution from tumor cells in HNSCC compared to other cancers. However, activated lymphocytes and impaired DNA clearance in inflammatory settings may also play a role^36,57–59^. The association of high cfDNA and NLR with poorer survival likely reflects the convergence of tumor progression and immune dysregulation, reinforcing the need for integrated immune profiling over single-marker approaches.

Taken together, these findings suggest that rmHNSCC subsets with dismal outcomes on immune checkpoint inhibition as monotherapy may be identifiable by blood-based diagnostics. In particular, our analysis highlights sCD25, IL-6, and TCR clonality as the strongest predictors of overall survival in patients receiving PD-1 blockade. While promising, these findings require validation in larger, independent cohorts. Refining immunotherapeutic strategies for these patients, possibly through combination with chemotherapy or other anti-inflammatory agents, may overcome resistance mechanisms and ultimately improve outcomes in rmHNSCC.

## Methods

### Biomaterial from the FOCUS clinical trial

This study is based on participants of the FOCUS trial (NCT05075122)^60^, which was conducted at 10 centers in Germany under approval of the local ethics committees and in compliance with the Declaration of Helsinki. The study was approved by the ethics committee (EC) of the Medical Faculty of the Martin-Luther-University Halle-Wittenberg, the EC of Medical Faculty of the University of Leipzig, the EC of the Landesärztekammer Rheinland-Pfalz, the EC of the Medical Faculty of the RWTH Aachen, the EC of the Landesärztekammer Baden-Württemberg, the Landesamt für Gesundheit und Soziales (LAGeSo Berlin), the EC of the Ärztekammer Hamburg, the EC of the Medical Faculty of the University of Würzburg, the EC of the University Medicine of Greifswald and the EC of the Sächsischen Landesärztekammer. Primary outcome data for the tested intervention have been published^21^. All participants provided written informed consent. Patients enrolled in the FOCUS clinical trial (NCT05075122) donated 20 mL of peripheral blood, which was collected in STRECK cell-free DNA BCT tubes (STRECK, Cat. no. 218997), at baseline assessment and prior to the second pembrolizumab dose for translational research. Collection and analysis of blood samples and anonymized patient data were approved by the local ethics committees of the collection sites, in compliance with the Declaration of Helsinki and local regulations.

### Survival analysis

Kaplan–Meier estimates were calculated using Cox proportional hazards models with p-values derived from log-rank test and hazard ratios derived from Cox regressions. Missing values were excluded. All comparative tests should be considered exploratory. Statistical significance was defined as p < 0.05. All analyses and plots were generated with R (v4.3.3, R Core Team, 2024), RStudio (v2024.12.1), and packages survminer (v0.4.9)^61^ and survival (v3.5.8)^62^.

### Next-generation T cell receptor repertoire sequencing

Leukocytes were pelleted from STRECK cell-free DNA BCT tubes (STRECK, Cat. no 218997) and genomic DNA was isolated using the GenElute Mammalian Genomic DNA Miniprep Kit (Sigma-Aldrich, Taufkirchen, Germany, Cat. no. G1N70-1KT) according to the manufacturer’s instructions. To analyze blood immune cells throughout treatment, amplification of the T cell receptor beta chain (TRB) repertoire from circulating cells was performed as described elsewhere^25,29,63–67^. Sequencing and de-multiplexing were performed on the Illumina MiSeq platform (Illumina, San Diego, CA) with paired-end reads (2 × 301 cycles) at an average coverage of 80,000 reads per sample. Alignment of rearranged TRB loci was performed using MiXCR (v3.0.12) and its default reference library^68^. All analyses and data plotting were carried out using R (v4.3.3, R Core Team, 2024)^69^ and the package tcR (v2.3.2)^70^. We calculated TCR richness, clonality, and diversity as non-redundant but complementary metrics to assess repertoire architectures. Richness refers to the total number of unique TCR clonotypes. Clonality as indicator for potential tumor-reactive clonotype expansion or proxy for oligoclonal repertoire with limited breadth is calculated as 1-Pielou’s evenness^71^, which measures the dominance of clonotypes in a repertoire (0 refers to a maximally diverse TCR repertoire, 1 to a repertoire dominated by one clonotype). Shannon entropy was calculated to quantify TCR repertoire diversity by incorporating both the number of unique TCR clonotypes (richness) and their relative frequencies (evenness). It was computed using the formula ${{\rm{H}}}_{1}=-{\sum }_{i=1}^{N}{{\rm{p}}}_{{\rm{i}}}\,\log ({{\rm{p}}}_{{\rm{i}}})$, where p_i_ represents the proportional frequency of each clonotype^71^. Higher Shannon entropy values indicate a more diverse and evenly distributed polyclonal/polyreactive repertoire, while lower values suggest clonal dominance or restricted diversity. Student’s t-test was used to compare two groups, and ANOVA was used to evaluate multiple groups. All comparative tests should be considered exploratory. Statistical significance was defined as p < 0.05. The datasets generated in this study have been deposited in the European Nucleotide Archive (ENA, ID: PRJEB80902).

### cfDNA quantification

Isolation of cfDNA from blood plasma was performed using the QIAamp Circulating Nucleic Acid Kit (Qiagen, Hilden, Germany, Cat. no. 55114) and quantified using Qubit dsDNA high-sensitivity assay (Thermo Fisher Scientific, Waltham, USA, Cat. no. Q32854).

### Soluble factor analysis

Plasma cytokines and other soluble factors were quantified using the LEGENDplex Human Immune Checkpoint Panel (10-plex), Anti-Virus Response Panel (13-plex), and the Human Inflammation Panel (13-plex) (BioLegend, Cat. no. 740961, 741273, and 740809) according to the manufacturer’s instructions. Readout of the LEGENDplex assays was performed on a CytoFLEX flow cytometer (Beckman Coulter Life Science). Correlation of plasma levels was calculated using the R package corrplot (v.0.92) with R (v4.3.3) and RStudio (v2023.06.1).

### Prognostic modeling

Multivariate analysis was performed using a Cox proportional hazards model with LASSO regularization, implemented via the glmnet package (v4.1-8)^72^. A 3-fold cross-validation approach was applied to select optimal regularization parameter based on the concordance index (C-index). Features with non-zero coefficients at the optimal lambda were retained and used to refit an unpenalized Cox model for interpretability. Predicted risk scores from the final model were used to asses survival stratification and prognostic performance. Time-dependent ROC curves at 6, 12, 18, and 24 months were computed using the timeROC package (v0.4)^73^ and corresponding AUC values were reported.

## Supplementary information

Supplement combinepdf final

## Data Availability

Data are available in the European Nucleotide Archive (ENA, ID: PRJEB80902) or upon request.

## Code availability

No code has been generated for this study. All analyses were performed with the referenced custom packages.
